# Unraveling the Proteomic Landscape of Intestinal Epithelial Cell-Derived Exosomes in Mice

**DOI:** 10.3389/fphys.2022.773671

**Published:** 2022-02-23

**Authors:** Zhenyu Ding, Cuiyu Zhang, Baokun Zhang, Qin Li

**Affiliations:** ^1^Department of Orthopedics, Shanghai Jiao Tong University Affiliated Sixth People’s Hospital, Shanghai, China; ^2^Department of Physiology, School of Basic Medical Sciences, Cheeloo College of Medicine, Shandong University, Jinan, China

**Keywords:** exosomes, intestinal epithelial cells, proteomics, exosome proteins, ACE2

## Abstract

**Purpose:**

This study aimed to identify the biological functions of small intestine intestinal epithelial cell derived exosomes (IEC-Exos) and further distinguished the difference proteins in IEC-Exos between ileum and jejunum related to function of the digestive system and occurrence of several diseases.

**Materials and Methods:**

IECs of Male C57BL/6J mice were isolated. IEC-Exos were extracted from jejunum and ileum epithelial cell culture fluid by ultracentrifugation. In addition, isobaric tags for relative and absolute quantitation (iTRAQ) combined with liquid chromatography-tandem mass spectrometry (LC-MS/MS) were used to detect IEC-Exo proteins and conduct biological information analysis.

**Results:**

The results showed that compared with jejunum IEC-Exos from ileum IEC-Exos, there were 393 up-regulated proteins and 346 down-regulated proteins. IECs-Exos, especially derived from jejunum, were rich in angiotensin-converting enzyme 2 (ACE2). The highly expressed proteins from ileum IEC-Exos were mostly enriched in genetic information processing pathways, which mainly mediate the processes of bile acid transport, protein synthesis and processing modification. In contrast, the highly expressed proteins from jejunum IEC-Exos were mainly enriched in metabolic pathways involved in sugar, fatty acid, amino acid, drug, and bone metabolism, etc. The differentially expressed proteins between ileum and jejunum IEC-Exos were not only related to the function of the digestive system but also closely related to the occurrence of infectious diseases, endocrine diseases and osteoarthritis, etc.

**Conclusion:**

IEC-Exos there were many differentially expressed proteins between ileum and jejunum, which played different roles in regulating intestinal biological functions. ACE2, the main host cell receptor of SARS-CoV-2, was highly expressed in IEC-Exos, which indicated that IEC-Exos may be a potential route of SARS-CoV-2 infection.

## Introduction

Intestinal epithelial cells (IECs), which have the characteristics of continuous migration, differentiation, and renewal, play an important role in maintaining intestinal function. As an organ involved in nutrient digestion and absorption, microbial defense, and endocrine functions, the intestine has important physiological functions, such as nutrient absorption, secretion, and transport. In addition, the intestine is also involved in drug metabolism, energy metabolism, endocrine responses, oxidative stress, and immune defense mechanisms (Peterson and Artis, 2014; Kiela and Ghishan, 2016; Satsu, 2017). The occurrence and development of clinical diabetes, atherosclerosis, intestinal inflammation, neurodegenerative diseases, and osteoarthritis are all correlated with intestinal epithelial dysfunction (de Wit et al., 2008; Gribble and Reimann, 2019; Tajik et al., 2020). Angiotensin-converting enzyme 2 (ACE2), which is highly expressed on IECs, is a receptor for severe acute respiratory syndrome coronavirus 2 (SARS-CoV-2)-infected cells and has an important effect on the occurrence and development of coronavirus disease 2019 (COVID-19) (Lamers et al., 2020; Ziegler et al., 2020). Recent studies confirmed that the exosomes are important intercellular communication vehicles, which play key role in cell biology. However, Intestinal epithelial cell derived exosomes (IEC-Exos) have not been studied in detail. Studies have shown that the biological function of IECs is closely related to their paracrine exosomes. Exosomes selectively enrich biologically active components derived from parent cells, including nucleic acids, proteins, lipids, amino acids, and metabolites, which are the basis for information and material exchange between cells. Once ingested by target cells, exosomes can regulate a variety of biological behaviors, such as gene expression and survival, proliferation, and migration of cells (Kalluri and LeBleu, 2020). IEC-Exos play pivotal roles in regulating intestinal antigen presentation, intestinal inflammation, intestinal homeostasis and systemic immune responses (van Niel and Heyman, 2002; Zhang H. et al., 2019). High-fat-induced IEC-Exos are involved in the occurrence and development of obesity, diabetes, and vascular complications (Xia et al., 2019). Many studies have demonstrated that exosomes play pivotal roles in the transmission of viruses and aggravation of adverse reactions and have potential application value in preventing viral infections (Hassanpour et al., 2020). Nevertheless, there are no reports on IEC-Exos in SARS-CoV-2 infection. At the same time, many significant biological functions, and mechanisms of IEC-Exos have not been fully elucidated.

The jejunum and ileum are the main components of the small intestine. The functional differences of exosomes derived from the jejunum and ileum are poorly understood. As a high-throughput screening method, proteomics has attracted increasing attention in the study of the protein components and biological effects of exosomes. In this study, extracted mouse jejunum and ileum IEC-Exos were identified by transmission electron microscopy, western blotting, and nanoparticle tracking analysis. For the first time, differentially expressed proteins of IEC-Exos were detected and compared through isobaric tags for relative and absolute quantitation (iTRAQ) combined with liquid chromatography-tandem mass spectrometry (LC-MS/MS). Gene Ontology (GO), Kyoto Encyclopedia of Genes and Genomes (KEGG) and protein–protein interaction (PPI) bioinformatics analyses were used to explore the function, distribution and related signaling pathways of differentially expressed proteins from ileum and jejunum IEC-Exos. Our study provides a basis for better understanding the function of IECs and for performing further research on the diagnosis and treatment of related diseases.

## Materials and Methods

### Cell Culture

Twenty-four male C57BL/6J mice (six mice per group) were sacrificed *via* cervical dislocation. The abdominal cavity of mice was opened, and the jejunum and ileum were gently cut out. The tissue was placed in a culture dish filled with cold normal saline. Then, the surplus mesenteric adipose tissue was gently removed, and the intestine was rinsed with cold saline. Next, the small intestine was cut longitudinally, and the stool was gently scraped off with a coverslip. The tissue was cut into small pieces (approximately 2 mm) and placed in a 50 mL centrifuge tube. DTT (30 ml, 10 mmol/L) was added to the tube and shaken for 5 min to remove mucus. After removing mucus, the tissue was transferred to a tube containing 30 mL 8 mmol/L EDTA and shaken for 30 min at 4°C. The EDTA was discarded, and 30 ml DPBS was added to a 50 mL centrifuge tube. The mixture was blown evenly with a 10 mL pipette, and the supernatant was collected. The above steps were repeated approximately six times until the supernatant became clear. The supernatant was mixed and centrifuged at 300 *g* for 5 min at 4°C. The supernatant was discarded, and the precipitate was collected. The precipitate was mainly composed of IECs. IECs of the jejunum and ileum were cultured with PBS, placed in a cell incubator at 37°C and 5% CO_2_, and cultured for 15 min. The culture fluid was collected to extract exosomes.

### Exosome Purification

Exosomes were isolated by differential centrifugation. In short, three-step centrifugation at 300 × *g* for 10 min, 2000 × *g* for 10 min, and 10,000 × *g* for 10 min were used to separate living cells, possible apoptotic bodies and large cell fragments from the culture fluid. Then, it was centrifuged at 100,000 × *g* for 70 min to collect EVs-containing pellets. After washing with phosphate buffered saline (PBS), ultracentrifugation was centrifuged at 100,000 × *g* for 70 min with a Beckman 32Ti rotor. The pellet was resuspended in PBS to obtain an IEC-Exo suspension of the jejunum and ileum. The size and particle number of EVs were characterized by electron microscope and nanoparticle tracking analysis (NTA).

### Nanoparticle Tracking Analysis

The exosome samples were first diluted to a final dilution of 1:5000 in filtered sterile PBS. The particle size and concentration distribution of the IEC-Exos were measured by NTA (ZetaVIEWS/N 17-310, PARTICLE METRIX, Meerbusch, Germany) according to the manufacturer’s instructions.

### Transmission Electron Microscopy

Twenty microliters of the exosome suspension was added to 2 nm copper grids, placed at room temperature for 3 min, and stained with a 3% phosphotungstic acid solution. The samples were then visualized using a transmission electron microscope (Tecnai G2 spirit Biotwin, FEI, Hillsboro, OR, United States).

### Western Blotting Analysis

The collected IEC-Exos were fully lysed by adding RIPI and a protease inhibitor (100:1). The lysis solution was centrifuged at 12,000 rpm for 20 min at 4°C. The supernatant was collected and mixed with 5x loading buffer. The mixture was placed in a heater for 10 min at 100°C to fully denature the protein. The expression of TSG 101 (Santa Cruz, sc-7964, dilution rate 1:500) and CD 81 (Santa Cruz, sc-166029, dilution rate 1:500) was detected by western blot analysis.

### Protein Extraction and Digestion

Frozen IEC-Exo samples from the ileum group and jejunum group were removed (four samples for each group, six mice per sample). The samples were homogenized with a hand-held homogenizer in the presence of lysate buffer on ice and then ultrasonically processed. The resulting solutions were centrifuged to remove any pellets and precipitates. A bicinchoninic acid assay (BCA) was performed to determine the protein concentration of every sample. Twenty micrograms of each sample of the same group were mixed together. Then, six volumes of acetone were added to each obtained group supernatant, and the mixed solutions were placed at –20°C overnight. After centrifugation, the supernatants were removed. The precipitated proteins were washed with acetone and resuspended in 500 mM triethylammonium bicarbonate/6 M guanidine hydrochloride (TEAB). BCA was performed to determine the protein concentration. A filter-aided sample preparation (FASP) strategy described in a previous report (Wisniewski et al., 2009) was performed for subsequent sample preparation. Briefly, 200 μg of protein from each group was reduced, alkylated and digested in centrifugal units. After digestion at 37°C overnight, the peptide solutions were centrifuged, and the filtrates were collected. Sequentially, the digested peptides were dried by vacuum centrifugation and stored at –80°C until further use.

### Isobaric Tags for Relative and Absolute Quantitation Labeling

One hundred micrograms samples from each group were labeled using a Reagent-8-plex Multiplex Kit according to the manufacturer’s instructions (AB Sciex, Darmstadt, Germany). The four ileum samples were labeled with reagents 113, 114, 117, 118 and the four jejunum samples were labeled with reagents 115, 116, 119 and 121. After resuspension in dissolution buffer, the digested peptides from each sample were incubated with a specific iTRAQ tag for 3 h at room temperature. The labeled samples were equally mixed and dried under a SpeedVac. After resuspension in 30 μL of 20 mM ammonium formate, 20 μL of the pooled sample was prefractionated by high-pH reverse-phase liquid chromatography using an ACQUITY UPLC H-Class Bio HPLC system (Thermo Scientific, Waltham, MA, United States), and finally, 10 consolidated fractions were acquired. Labeled peptides in each fraction were dried and redissolved in 30 μL 2% acetonitrile/0.1% formic acid for LC-MS/MS analysis.

### Liquid Chromatography-Tandem Mass Spectrometry

The labeled peptide mixtures were separated using an EASY-nLC 1200 system (Thermo Scientific, United States). The peptides were separated on a PepMap100 RSLC C18, 2 μm, 75 μm × 15 cm analytic column using a 110-min mobile phase gradient from 5 to 30%. Mass spectra were recorded on an Orbitrap Exploris 480 mass spectrometer conFigured with a Nano-ESI source (Thermo Scientific, United States). Full scan MS spectra were acquired in the m/z range of 350–1600 at a resolution of 70,000. The top 10 precursors were selected for high-energy collision-induced dissociation (HCD) with a collision energy of 35%, and the product ions were detected at a resolution of 17,500 in data-dependent acquisition mode.

### Isobaric Tags for Relative and Absolute Quantitation Data Analysis

The raw data were searched against the UniProt database using MASCOT2.3.2 (Thermo Scientific, United States; Matrix Science, London, United Kingdom), a search engine built in the Proteome Discoverer 1.4 software suite. Protein level changes were compared between paired ileum IEC-Exos and jejunum IEC-Exos, and differentially expressed proteins (fold change ≥ 1.3 or ≤0.769 and *P* < 0.05) were defined. The data were analyzed by Bohao Biotechnologies Co., Ltd. (Shanghai, China).

### Bioinformatic Analysis

Gene Ontology, KEGG and PPI bioinformatics analyses were performed on the differentially expressed proteins between the ileum and jejunum IEC-Exos. Enrichment analysis of the GO terms, including molecular function (MF), biological process (BP) and cellular component (CC), and KEGG pathway analysis of the dysregulated proteins were performed using the DAVID web server. Fisher’s exact test was used to test the significance of the overlap between various gene sets, and P values < 0.05 were considered significant. The smallest 15 *P*-values or the 30 items with the smallest *P*-values between the up- or down-regulated differentially expressed proteins were selected to draw bar plots or dotplots. The PPI network was analyzed with the String database^1^ and visualized with Cytoscape software.

## Results

### Intestinal Epithelial Cell Derived Exosomes Identification

**FIGURE 1:**
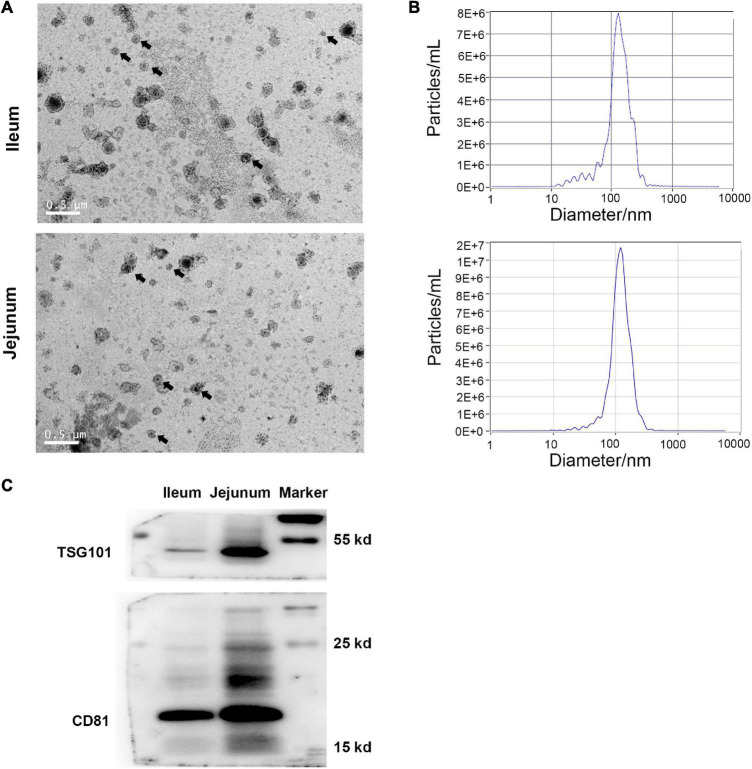
Identification of IEC-Exos from ileum and jejunum. **(A)** Transmission electron microscopic observation (scale = 0.5 μm), arrows indicated the typical exosomes. **(B)** Nanoparticle tracking analyzer Size Distribution. **(C)** Immunoblot of CD81 and TSG101 in IEC-Exos.

Under transmission electron microscopy, both ileum and jejunum IEC-Exos presented as round or elliptical vesicles with clear double-layer membrane structures (Figure 1A). The results of nanoparticle tracking analysis showed that the concentration of ileum IEC-Exos was 1.0E + 8 particles/mL, and the diameter of IEC-Exos was mainly concentrated at 30–130 nm. The concentration of jejunum IEC-Exos was 1.2E + 8 particles/mL with a diameter of 30–120 nm (Figure 1B). In addition, the exosomal intracellular proteins CD81 and TSG101 were also detected by western blotting (Figure 1C).

### Intestinal Epithelial Cell Derived Exosomes Proteins Quantification

**FIGURE 2:**
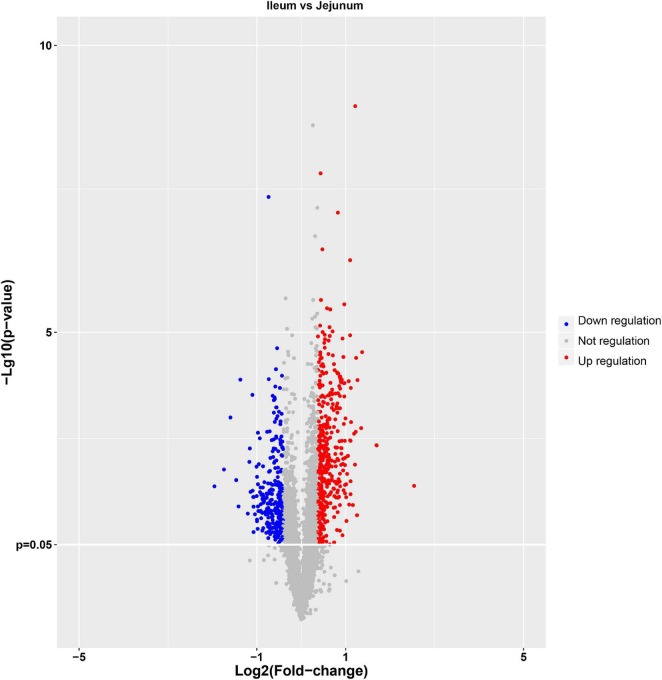
Volcano map of differentially expressed proteins. The abscissa represents the fold change (log2 value) of differentially expressed proteins, and the ordinate represents the *P*-value (–log10). Gray points represent proteins with insignificant differences, red points represent up-regulated proteins, and the blue points represent down-regulated proteins.

The protein quantification results showed that 4140 proteins were identified in IEC-Exos, and compared with jejunum IEC-Exos, there were 739 differentially expressed proteins(fold change ≥ 1.3 or ≤0.769 and *P* < 0.05) from ileum IEC-Exos, including 393 up-regulated proteins and 346 down-regulated proteins. Moreover, there was no difference in the expression of 3401 proteins between the two types of IEC-Exos (Figure 2). Ribosomal proteins were the majority of the 40 up-regulated proteins (Table 1). Solute carrier (SLC) transporters carbohydrates, amino acids, nucleic acids, ions and drugs were the majority of the down-regulated proteins (Table 2 and Schedule 1). However, Slc10a2 (solute carrier family 10 member 2), which is related to the uptake of intestinal bile acids in the distal ileum, was significantly up-regulated in IEC-Exos (Table 1 and Schedule 1). Based on previously reported cell type–specific markers and intestinal single-cell sequencing results(Haber et al., 2017; Wang Y. et al., 2020), we confirmed that the IEC-Exos we isolated were mainly derived from eight known intestinal epithelial cells, including enterocyte cells, goblet cells, Paneth cells, enteroendocrine cells, tuft cells, progenitor cells, transit amplifying cells (TACs) and stem cells (Schedule 2).

**TABLE 1:** Top 40 up-regulated protein information.

No.	Accession	Gene symbol	Description	Score	Fold change (ileum/jejunum)	*P*-value	Expression
1	P70172	Slc10a2	Ileal sodium/bile acid cotransporter	24.88	2.734	0.000	Up
2	P51162	Fabp6	Gastrotropin	819.27	2.595	0.005	Up
3	P62862	Fau	40S ribosomal protein S30	29.05	2.590	0.001	Up
4	Q45VN2	Defa20	Alpha-defensin 20	393.21	2.516	0.001	Up
5	P14115	Rpl27a	60S ribosomal protein L27a	440.27	2.153	0.000	Up
6	P84104	Srsf3	Serine/arginine-rich splicing factor 3	262.16	2.105	0.004	Up
7	Q9D7L8	Tmigd1	Transmembrane and immunoglobulin domain-containing protein 1	42.03	2.081	0.000	Up
8	Q9D1R9	Rpl34	60S ribosomal protein L34	227.64	2.071	0.001	Up
9	P62984	Uba52	Ubiquitin-60S ribosomal protein L40	326.89	2.006	0.019	Up
10	Q9Z130	Hnrnpdl	Heterogeneous nuclear ribonucleoprotein D-like	167.32	2.002	0.000	Up
11	P62754	Rps6	40S ribosomal protein S6	1661.69	1.998	0.001	Up
12	Q8BG05	Hnrnpa3	Heterogeneous nuclear ribonucleoprotein A3	855.48	1.984	0.010	Up
13	Q3TTY0	Plb1	Phospholipase B1, membrane-associated	926.16	1.962	0.007	Up
14	Q9CS00	Cactin	Cactin	33.31	1.961	0.046	Up
15	Q6PFR5	Tra2a	Transformer-2 protein homolog alpha	235.15	1.938	0.005	Up
16	P62270	Rps18	40S ribosomal protein S18	853.50	1.917	0.000	Up
17	P63276	Rps17	40S ribosomal protein S17	363.00	1.916	0.005	Up
18	P47963	Rpl13	60S ribosomal protein L13	730.79	1.871	0.000	Up
19	P09405	Ncl	Nucleolin	1192.56	1.866	0.000	Up
20	Q9WV02	Rbmx	RNA-binding motif protein, X chromosome	268.55	1.851	0.014	Up
21	P56959	Fus	RNA-binding protein FUS	304.31	1.849	0.001	Up
22	P83882	Rpl36a	60S ribosomal protein L36a	134.14	1.841	0.000	Up
23	P62852	Rps25	40S ribosomal protein S25	154.37	1.840	0.000	Up
24	P62264	Rps14	40S ribosomal protein S14	669.66	1.816	0.010	Up
25	Q8K1N4	Spats2	Spermatogenesis-associated serine-rich protein 2	24.89	1.812	0.004	Up
26	O55142	Rpl35a	60S ribosomal protein L35a	248.54	1.810	0.006	Up
27	Q9CZX8	Rps19	40S ribosomal protein S19	517.65	1.807	0.029	Up
28	Q9CXW4	Rpl11	60S ribosomal protein L11	261.68	1.806	0.011	Up
29	Q61470	Cd37	Leukocyte antigen CD37	27.41	1.801	0.049	Up
30	P70245	Ebp	3-beta-hydroxysteroid-Delta(8), Delta(7)-isomerase	49.48	1.793	0.002	Up
31	P70372	Elavl1	ELAV-like protein 1	417.63	1.793	0.000	Up
32	Q8VEK3	Hnrnpu	Heterogeneous nuclear ribonucleoprotein U	1053.62	1.790	0.002	Up
33	Q61545	Ewsr1	RNA-binding protein EWS	111.66	1.788	0.000	Up
34	P41105	Rpl28	60S ribosomal protein L28	425.77	1.785	0.005	Up
35	Q99L45	Eif2s2	Eukaryotic translation initiation factor 2 subunit 2	202.05	1.781	0.000	Up
36	Q6PDM2	Srsf1	Serine/arginine-rich splicing factor 1	384.95	1.781	0.004	Up
37	Q99020	Hnrnpab	Heterogeneous nuclear ribonucleoprotein A/B	593.69	1.781	0.001	Up
38	Q6ZWV7	Rpl35	60S ribosomal protein L35	177.35	1.780	0.002	Up
39	P62849	Rps24	40S ribosomal protein S24	595.00	1.747	0.011	Up
40	Q62189	Snrpa	U1 small nuclear ribonucleoprotein A	251.93	1.741	0.000	Up

**TABLE 2:** Top 40 down-regulated protein information.

No.	Accession	Gene symbol	Description	Score	Fold change (ileum/jejunum)	*P*-value	Expression
1	Q8R0I0	Ace2	Angiotensin-converting enzyme 2	5441.54	0.685	0.008	Down
2	O88627	Slc28a2	Sodium/nucleoside cotransporter 2	93.84	0.680	0.014	Down
3	Q3U9N9	Slc16a10	Monocarboxylate transporter 10	167.24	0.668	0.019	Down
4	Q0VG18	Smim24	Small integral membrane protein 24	682.87	0.668	0.002	Down
5	Q60931	Vdac3	Voltage-dependent anion-selective channel protein 3	448.05	0.665	0.047	Down
6	P48758	Cbr1	Carbonyl reductase [NADPH] 1	311.56	0.654	0.026	Down
7	Q9QXW9	Slc7a8	Large neutral amino acids transporter small subunit 2	92.10	0.645	0.033	Down
8	Q9JIL4	Pdzk1	Na(+)/H(+) exchange regulatory cofactor NHE-RF3	1923.69	0.640	0.009	Down
9	Q75N73	Slc39a14	Zinc transporter ZIP14	84.74	0.640	0.010	Down
10	Q9DCN2	Cyb5r3	NADH-cytochrome b5 reductase 3	623.13	0.639	0.030	Down
11	O09131	Gsto1	Glutathione S-transferase omega-1	273.14	0.638	0.017	Down
12	Q9JHE3	Asah2	Neutral ceramidase	1167.66	0.638	0.004	Down
13	Q8VDB9	Slc6a20a	Sodium- and chloride-dependent transporter XTRP3A	40.67	0.635	0.010	Down
14	Q8K0E3	Slc5a11	Sodium/myo-inositol cotransporter 2	365.10	0.622	0.000	Down
15	P09470	Ace	Angiotensin-converting enzyme	1955.22	0.618	0.003	Down
16	O08601	Mttp	Microsomal triglyceride transfer protein large subunit	5405.93	0.613	0.026	Down
17	Q8C3K6	Slc5a1	Sodium/glucose cotransporter 1	785.16	0.606	0.007	Down
18	Q61391	Mme	Neprilysin	3797.97	0.597	0.006	Down
19	Q9WV38	Slc2a5	Solute carrier family 2, facilitated glucose transporter member 5	56.26	0.596	0.009	Down
20	P34914	Ephx2	Bifunctional epoxide hydrolase 2	548.11	0.584	0.038	Down
21	Q8CIW6	Slc26a6	Solute carrier family 26 member 6	639.88	0.579	0.013	Down
22	Q64459	Cyp3a11	Cytochrome P450 3A11	395.46	0.576	0.030	Down
23	Q9Z0S1	Bpnt1	3’(2’),5’-bisphosphate nucleotidase 1	865.65	0.573	0.017	Down
24	P24822	Iap	Intestinal-type alkaline phosphatase	2108.12	0.573	0.000	Down
25	Q6DYE8	Enpp3	Ectonucleotide pyrophosphatase/phosphodiesterase family member 3	1258.82	0.572	0.001	Down
26	Q91WG0	Ces2c	Acylcarnitine hydrolase	671.42	0.570	0.004	Down
27	P14246	Slc2a2	Solute carrier family 2, facilitated glucose transporter member 2	342.70	0.567	0.019	Down
28	P12791	Cyp2b10	Cytochrome P450 2B10	214.30	0.567	0.026	Down
29	Q08652	Rbp2	Retinol-binding protein 2	2305.26	0.550	0.014	Down
30	Q8QZR3	Ces2a	Pyrethroid hydrolase Ces2a	453.70	0.549	0.003	Down
31	P32020	Scp2	Non-specific lipid-transfer protein	130.87	0.543	0.002	Down
32	P63254	Crip1	Cysteine-rich protein 1	32.45	0.526	0.007	Down
33	P06728	Apoa4	Apolipoprotein A-IV	493.69	0.525	0.007	Down
34	O08691	Arg2	Arginase-2, mitochondrial	387.91	0.522	0.013	Down
35	Q04447	Ckb	Creatine kinase B-type	784.18	0.513	0.018	Down
36	Q8BK48	Ces2e	Pyrethroid hydrolase Ces2e	896.89	0.503	0.006	Down
37	P12710	Fabp1	Fatty acid-binding protein, liver	2016.15	0.497	0.002	Down
38	Q08423	Tff1	Trefoil factor 1	39.09	0.484	0.002	Down
39	P00329	Adh1	Alcohol dehydrogenase 1	295.83	0.387	0.011	Down
40	Q8K1F9	Lctl	Lactase-like protein	27.66	0.384	0.004	Down

**SCHEDULE 1:** Solute carrier transporter protein information.

No.	Accession	Gene symbol	Description	Score	Fold change (ileum/jejunum)	*P*-value	Expression
1	P70172	Slc10a2	Ileal sodium/bile acid cotransporter	24.88	2.734	0.000	Up
2	Q9R0M8	Slc35a2	UDP-galactose translocator	65.23	1.390	0.001	Up
3	Q8R000	Slc51a	Organic solute transporter subunit alpha	182.08	1.345	0.006	Up
4	Q762D5	Slc35d2	UDP-*N*-acetylglucosamine/UDP-glucose/GDP-mannose transporter	20.13	1.345	0.002	Up
5	Q922Q5	Slc35b3	Adenosine 3′-phospho 5′-phosphosulfate transporter 2	25.75	1.334	0.003	Up
6	D3Z5L6	Slc18b1	MFS-type transporter SLC18B1	34.59	1.314	0.053	Not
7	P53986	Slc16a1	Monocarboxylate transporter 1	208.23	0.764	0.028	Down
8	Q8K4D3	Slc36a1	Proton-coupled amino acid transporter 1	54.68	0.758	0.013	Down
9	Q9JIP7	Slc15a1	Solute carrier family 15 member 1	1489.24	0.756	0.022	Down
10	O35488	Slc27a2	Very long-chain acyl-CoA synthetase	233.95	0.747	0.091	Not
11	Q3UVU3	Slc30a10	Zinc transporter 10	75.21	0.735	0.083	Not
12	Q5DTL9	Slc4a10	Sodium-driven chloride bicarbonate exchanger	39.14	0.734	0.025	Down
13	P32037	Slc2a3	Solute carrier family 2, facilitated glucose transporter member 3	25.83	0.734	0.227	Not
14	Q8VEM8	Slc25a3	Phosphate carrier protein, mitochondrial	250.32	0.732	0.000	Down
15	Q9Z2J0	Slc23a1	Solute carrier family 23 member 1	26.97	0.721	0.014	Down
16	G3 × 939	Slc9a3	Sodium/hydrogen exchanger 3	395.76	0.716	0.012	Down
17	Q9D687	Slc6a19	Sodium-dependent neutral amino acid transporter B(0)AT1	263.89	0.710	0.005	Down
18	P70441	Slc9a3r1	Na(+)/H(+) exchange regulatory cofactor NHE-RF1	1535.24	0.699	0.000	Down
19	P10852	Slc3a2	4F2 cell-surface antigen heavy chain	1413.74	0.694	0.013	Down
20	Q8BTY2	Slc4a7	Sodium bicarbonate cotransporter 3	254.31	0.692	0.031	Down
21	Q7TML3	Slc35f2	Solute carrier family 35 member F2	50.19	0.691	0.000	Down
22	Q9EPR4	Slc23a2	Solute carrier family 23 member 2	53.34	0.685	0.045	Down
23	O88627	Slc28a2	Sodium/nucleoside cotransporter 2	93.84	0.680	0.014	Down
24	Q3U9N9	Slc16a10	Monocarboxylate transporter 10	167.24	0.668	0.019	Down
25	Q9QXW9	Slc7a8	Large neutral amino acids transporter small subunit 2	92.10	0.645	0.033	Down
26	Q75N73	Slc39a14	Zinc transporter ZIP14	84.74	0.640	0.010	Down
27	Q8VDB9	Slc6a20a	Sodium- and chloride-dependent transporter XTRP3A	40.67	0.635	0.010	Down
28	Q8K0E3	Slc5a11	Sodium/myo-inositol cotransporter 2	365.10	0.622	0.000	Down
29	Q8C3K6	Slc5a1	Sodium/glucose cotransporter 1	785.16	0.606	0.007	Down
30	Q9WV38	Slc2a5	Solute carrier family 2, facilitated glucose transporter member 5	56.26	0.596	0.009	Down
31	Q8CIW6	Slc26a6	Solute carrier family 26 member 6	639.88	0.579	0.013	Down
32	P14246	Slc2a2	Solute carrier family 2, facilitated glucose transporter member 2	342.70	0.567	0.019	Down

**SCHEDULE 2:** Marker protein information of intestinal cell cluster.

No.	Accession	Gene symbol	Description	Score	Fold change (ileum/jejunum)	*P*-value	Expression	Cluster
1	P51162	Fabp6	Gastrotropin	819.27	2.595	0.005	Up	Enterocyte
2	Q9D7L8	Tmigd1	Transmembrane and immunoglobulin domain-containing protein 1	42.03	2.081	0.000	Up	Enterocyte
3	P28825	Mep1a	Meprin A subunit alpha	611.66	1.297	0.000	Not	Enterocyte
4	Q80WK2	Slc51b	Organic solute transporter subunit beta	32.54	1.296	0.015	Not	Enterocyte
5	Q7M758	Naaladl1	*N*-acetylated-alpha-linked acidic dipeptidase-like protein	3050.06	1.259	0.020	Not	Enterocyte
6	P04441	Cd74	H-2 class II histocompatibility antigen gamma chain	121.63	1.224	0.004	Not	Enterocyte
7	Q00623	Apoa1	Apolipoprotein A-I	345.33	1.170	0.010	Not	Enterocyte
8	P29391	Ftl1	Ferritin light chain 1	224.74	1.149	0.011	Not	Enterocyte
9	Q9Z2A7	Dgat1	Diacylglycerol *O*-acyltransferase 1	299.61	1.076	0.959	Not	Enterocyte
10	Q9D312	Krt20	Keratin, type I cytoskeletal 20	604.04	1.059	0.027	Not	Enterocyte
11	P09528	Fth1	Ferritin heavy chain	197.16	1.045	0.597	Not	Enterocyte
12	Q62159	Rhoc	Rho-related GTP-binding protein RhoC	433.22	1.038	0.002	Not	Enterocyte
13	P19001	Krt19	Keratin, type I cytoskeletal 19	1029.77	1.028	0.214	Not	Enterocyte
14	Q9QYZ9	Prss30	Serine protease 30	48.48	1.003	0.164	Not	Enterocyte
15	P21460	Cst3	Cystatin-C	47.14	0.976	0.847	Not	Enterocyte
16	Q8K3K7	Agpat2	1-acyl-sn-glycerol-3-phosphate acyltransferase beta	143.84	0.975	0.903	Not	Enterocyte
17	E9Q414	Apob	Apolipoprotein B-100	2953.79	0.971	0.690	Not	Enterocyte
18	P31428	Dpep1	Dipeptidase 1	959.01	0.960	0.381	Not	Enterocyte
19	P22599	Serpina1b	Alpha-1-antitrypsin 1-2	142.90	0.933	0.516	Not	Enterocyte
20	Q8JZQ5	Aoc1	Amiloride-sensitive amine oxidase	285.68	0.927	0.016	Not	Enterocyte
21	O70404	Vamp8	Vesicle-associated membrane protein 8	62.32	0.923	0.013	Not	Enterocyte
22	Q9ET47	Espn	Espin	70.78	0.907	0.057	Not	Enterocyte
23	P97449	Anpep	Aminopeptidase N	20449.97	0.887	0.013	Not	Enterocyte
24	P01887	B2m	Beta-2-microglobulin	573.23	0.884	0.119	Not	Enterocyte
25	E9Q7P9	Cdhr2	Cadherin-related family member 2	756.20	0.858	0.018	Not	Enterocyte
26	Q8VHF2	Cdhr5	Cadherin-related family member 5	1755.79	0.850	0.004	Not	Enterocyte
27	P33622	Apoc3	Apolipoprotein C-III	72.17	0.845	0.402	Not	Enterocyte
28	P18242	Ctsd	Cathepsin D	70.48	0.823	0.511	Not	Enterocyte
29	Q9QXA6	Slc7a9	b(0, +)-type amino acid transporter 1	54.43	0.806	0.082	Not	Enterocyte
30	O88329	Myo1a	Unconventional myosin-Ia	4066.40	0.771	0.000	Not	Enterocyte
31	Q8VDN2	Atp1a1	Sodium/potassium-transporting ATPase subunit alpha-1	19959.97	0.770	0.036	Not	Enterocyte
32	Q9Z2V4	Pck1	Phosphoenolpyruvate carboxykinase, cytosolic [GTP]	163.75	0.768	0.018	Down	Enterocyte
33	P55050	Fabp2	Fatty acid-binding protein, intestinal	1541.20	0.740	0.001	Down	Enterocyte
34	Q91Y97 (0.718)	Aldob	Fructose-bisphosphate aldolase B	3717.86	0.718	0.008	Down	Enterocyte
35	P97328	Khk	Ketohexokinase	627.48	0.716	0.008	Down	Enterocyte
36	Q9D687	Slc6a19	Sodium-dependent neutral amino acid transporter B(0)AT1	263.89	0.710	0.005	Down	Enterocyte
37	O09051	Guca2b	Guanylate cyclase activator 2B	80.83	0.685	0.058	Not	Enterocyte
38	Q8R0I0	Ace2	Angiotensin-converting enzyme 2	5441.54	0.685	0.008	Down	Enterocyte
39	Q0VG18	Smim24	Small integral membrane protein 24 OS	682.87	0.668	0.002	Down	Enterocyte
40	P09470	Ace	Angiotensin-converting enzyme	1955.22	0.618	0.003	Down	Enterocyte
41	P63254	Crip1	Cysteine-rich protein 1	32.45	0.526	0.007	Down	Enterocyte
42	P06728	Apoa4	Apolipoprotein A-IV OS	493.69	0.525	0.007	Down	Enterocyte
43	P12710	Fabp1	Fatty acid-binding protein, liver	2016.15	0.497	0.002	Down	Enterocyte
44	P14115	Rpl27a	60S ribosomal protein L27a	440.27	2.153	0.000	Up	Globlet
45	P62281	Rps11	40S ribosomal protein S11	517.60	1.697	0.001	Up	Globlet
46	P47964	Rpl36	60S ribosomal protein L36	112.08	1.569	0.003	Up	Globlet
47	Q9JJI8	Rpl38	60S ribosomal protein L38	58.64	1.563	0.002	Up	Globlet
48	Q9D7Z6	Clca1	Calcium-activated chloride channel regulator 1	4448.48	1.392	0.011	Up	Globlet
49	Q91VT8	Smim14	Small integral membrane protein 14	34.71	1.378	0.001	Up	Globlet
50	O88312	Agr2	Anterior gradient protein 2 homolog	196.89	1.281	0.003	Not	Globlet
51	P62274	Rps29	40S ribosomal protein S29	50.38	1.211	0.007	Not	Globlet
52	Q9ERI2	Rab27a	Ras-related protein Rab-27A	41.66	1.197	0.001	Not	Globlet
53	Q8K0C5	Zg16	Zymogen granule membrane protein 16	735.06	1.164	0.180	Not	Globlet
54	P62748	Hpcal1	Hippocalcin-like protein 1	38.78	1.152	0.345	Not	Globlet
55	P97805	Fam3d	Protein FAM3D	555.06	1.149	0.017	Not	Globlet
56	P13020	Gsn	Gelsolin	501.63	1.137	0.008	Not	Globlet
57	Q80YN3	Bcas1	Breast carcinoma-amplified sequence 1 homolog	35.47	1.107	0.074	Not	Globlet
58	Q9D8C2	Tspan13	Tetraspanin-13	137.69	1.103	0.028	Not	Globlet
59	O88310	Itln1	Intelectin-1a	177.88	1.099	0.050	Not	Globlet
60	P58771	Tpm1	Tropomyosin alpha-1 chain	136.14	1.070	0.157	Not	Globlet
61	Q91VW3	Sh3bgrl3	SH3 domain-binding glutamic acid-rich-like protein 3	20.79	1.055	0.365	Not	Globlet
62	Q62395	Tff3	Trefoil factor 3	286.15	1.055	0.365	Not	Globlet
63	P55012	Slc12a2	Solute carrier family 12 member 2	604.04	0.980	0.771	Not	Globlet
64	P05784	Krt18	Keratin, type I cytoskeletal 18	214.22	0.953	0.843	Not	Globlet
65	Q9D7T1	Rep15	Rab15 effector protein	28.61	0.952	0.178	Not	Globlet
66	P63323	Rps12	40S ribosomal protein S12	49.83	0.950	0.056	Not	Globlet
67	Q9JMD3	Stard10	START domain-containing protein 10	98.71	0.866	0.432	Not	Globlet
68	P09036	Spink1	Serine protease inhibitor Kazal-type 1	94.78	0.777	0.120	Not	Globlet
69	Q45VN2	Defa20	Alpha-defensin 20	393.21	2.516	0.001	Up	Peneth
70	Q8C1N8	Defa22	Alpha-defensin 22	315.95	1.524	0.001	Up	Peneth
71	Q5G865	Defa24	Alpha-defensin 24	71.26	1.515	0.003	Up	Peneth
72	P17897	Lyz1	Lysozyme C-1	517.86	1.301	0.003	Up	Peneth
73	P28309	Defa2	Alpha-defensin 2	49.47	1.220	0.049	Not	Peneth
74	Q64444	Ca4	Carbonic anhydrase 4	168.49	1.102	0.007	Not	Peneth
75	P50711	Defa13	Alpha-defensin 13	36.98	1.081	0.135	Not	Peneth
76	P26883	Fkbp1a	Peptidyl-prolyl *cis-trans* isomerase FKBP1A	179.65	0.949	0.031	Not	Peneth
77	P41731	Cd63	CD63 antigen	65.25	0.890	0.852	Not	Peneth
78	P08207	S100a10	Protein S100-A10	59.24	0.874	0.569	Not	Peneth
79	Q9D7S0	Lypd8	Ly6/PLAUR domain-containing protein 8	147.86	0.835	0.000	Not	Peneth
80	P50543	S100a11	Protein S100-A11	96.64	0.830	0.025	Not	Peneth
81	O09051	Guca2b	Guanylate cyclase activator 2B	80.83	0.685	0.058	Not	Peneth
82	Q9JLJ1	Selenok	Selenoprotein K	24.22	1.224	0.015	Not	Enteroendocrine
83	P07309	Ttr	Transthyretin	24.02	1.138	0.202	Not	Enteroendocrine
84	Q62186	Ssr4	Translocon-associated protein subunit delta	219.86	1.109	0.000	Not	Enteroendocrine
85	P16014	Chgb	Secretogranin-1	0.00	0.973	0.687	Not	Enteroendocrine
86	Q3UWA6	Gucy2c	Heat-stable enterotoxin receptor	182.61	0.961	0.467	Not	Enteroendocrine
87	Q9D0J8	Ptms	Parathymosin	35.09	0.800	0.010	Not	Enteroendocrine
88	Q08423	Tff1	Trefoil factor 1	39.09	0.484	0.002	Down	Enteroendocrine
89	P62754	Rps6	40S ribosomal protein S6	1661.69	1.998	0.001	Up	Progenitor
90	P62270	Rps18	40S ribosomal protein S18	853.50	1.917	0.000	Up	Progenitor
91	P63276	Rps17	40S ribosomal protein S17	363.00	1.916	0.005	Up	Progenitor
92	P47911	Rpl6	60S ribosomal protein L6	1149.18	1.732	0.001	Up	Progenitor
93	Q9CZM2	Rpl15	60S ribosomal protein L15	585.12	1.7	0.000	Up	Progenitor
94	P14131	Rps16	40S ribosomal protein S16	518.88	1.692	0.000	Up	Progenitor
95	P62983	Rps27a	Ubiquitin-40S ribosomal protein S27a	1180.02	1.645	0.000	Up	Progenitor
96	P62082	Rps7	40S ribosomal protein S7	493.66	1.560	0.000	Up	Progenitor
97	P61358	Rpl27	60S ribosomal protein L27	356.71	1.557	0.008	Up	Progenitor
98	P62918	Rpl8	60S ribosomal protein L8	1301.11	1.527	0.000	Up	Progenitor
99	P62717	Rpl18a	60S ribosomal protein L18a	804.60	1.524	0.001	Up	Progenitor
100	P14148	Rpl7	60S ribosomal protein L7	852.98	1.522	0.000	Up	Progenitor
101	P53026	Rpl10a	60S ribosomal protein L10a	842.42	1.514	0.004	Up	Progenitor
102	P47962	Rpl5	60S ribosomal protein L5	637.28	1.330	0.003	Up	Progenitor
103	P14206	Rpsa	40S ribosomal protein SA	284.21	0.957	0.094	Not	Progenitor
104	Q9JHC0	Gpx2	Glutathione peroxidase 2	79.25	0.909	0.548	Not	Progenitor
105	Q91VS7	Mgst1	Microsomal glutathione S-transferase 1	98.93	0.905	0.540	Not	Progenitor
106	P99028	Uqcrh	Cytochrome b-c1 complex subunit 6, mitochondrial	181.84	0.832	0.091	Not	Progenitor
107	Q60829	Ppp1r1b	Protein phosphatase 1 regulatory subunit 1B	55.03	0.831	0.164	Not	Progenitor
108	Q9CR84	Atp5g1	ATP synthase F(0) complex subunit C1, mitochondrial	43.52	0.775	0.006	Not	Progenitor
109	P10639	Txn	Thioredoxin	418.72	0.686	0.012	Down	Progenitor
110	P04184	Tk1	Thymidine kinase, cytosolic	0.00	1.110	0.037	Not	TA
111	P30681	Hmgb2	High mobility group protein B2	122.02	1.039	0.340	Not	TA
112	P17918	Pcna	Proliferating cell nuclear antigen	97.16	1.035	0.033	Not	TA
113	P63158	Hmgb1	High mobility group protein B1	148.21	0.979	0.094	Not	TA
114	P0C0S6	H2afz	Histone H2A.Z	185.48	0.892	0.091	Not	TA
115	P07607	Tyms	Thymidylate synthase	0.00	0.879	0.185	Not	TA
116	P54227	Stmn1	Stathmin	46.66	0.863	0.149	Not	TA
117	P26350	Ptma	Prothymosin alpha	418.82	0.779	0.076	Not	TA
118	P35979	Rpl12	60S ribosomal protein L12	1369.53	1.323	0.002	Up	Stem
119	P60229	Eif3e	Eukaryotic translation initiation factor 3 subunit E	467.06	1.288	0.005	Not	Stem
120	Q3UZZ4	Olfm4	Olfactomedin-4	131.23	1.037	0.012	Not	Stem
121	P55012	Slc12a2	Solute carrier family 12 member 2	604.04	0.980	0.771	Not	Stem
122	Q02013	Aqp1	Aquaporin-1	111.24	0.738	0.036	Down	Stem
123	Q91VM5	Rbmxl1	RNA binding motif protein, X-linked-like-1	283.41	1.601	0.037	Up	Tuft
124	Q9R0N0	Galk1	Galactokinase	32.33	1.408	0.009	Up	Tuft
125	P46735	Myo1b	Unconventional myosin-Ib	497.04	1.373	0.911	Not	Tuft
126	Q9R1Q6	Tmem176b	Transmembrane protein 176B	22.45	1.065	0.068	Not	Tuft
127	Q6WVG3	Kctd12	BTB/POZ domain-containing protein KCTD12	64.55	0.961	0.045	Not	Tuft
128	P05784	Krt18	Keratin, type I cytoskeletal 18	214.22	0.953	0.843	Not	Tuft
129	Q61152	Ptpn18	Tyrosine-protein phosphatase non-receptor type 18	26.20	0.943	0.204	Not	Tuft
130	P29351	Ptpn6	Tyrosine-protein phosphatase non-receptor type 6	72.24	0.937	0.138	Not	Tuft
131	Q9CPT0	Bcl2l14	Apoptosis facilitator Bcl-2-like protein 14	93.85	0.923	0.374	Not	Tuft
132	Q8CIH5	Plcg2	1-phosphatidylinositol 4,5-bisphosphate phosphodiesterase gamma-2	90.26	0.917	0.589	Not	Tuft
133	P47738	Aldh2	Aldehyde dehydrogenase, mitochondrial	188.39	0.874	0.000	Not	Tuft
134	Q61735	Cd47	Leukocyte surface antigen CD47	258.93	0.803	0.056	Not	Tuft
135	P08103	Hck	Tyrosine-protein kinase HCK	62.45	0.787	0.195	Not	Tuft
136	Q9ERG0	Lima1	LIM domain and actin-binding protein 1	480.76	0.703	0.001	Down	Tuft
137	Q60931	Vdac3	Voltage-dependent anion-selective channel protein 3	448.05	0.665	0.047	Down	Tuft
138	P00329	Adh1	Alcohol dehydrogenase 1	295.83	0.387	0.011	Down	Tuft

### Bioinformatics Analysis

#### Gene Ontology Analysis of Intestinal Epithelial Cell Derived Exosomes Proteins From the Ileum and Jejunum

**FIGURE 3:**
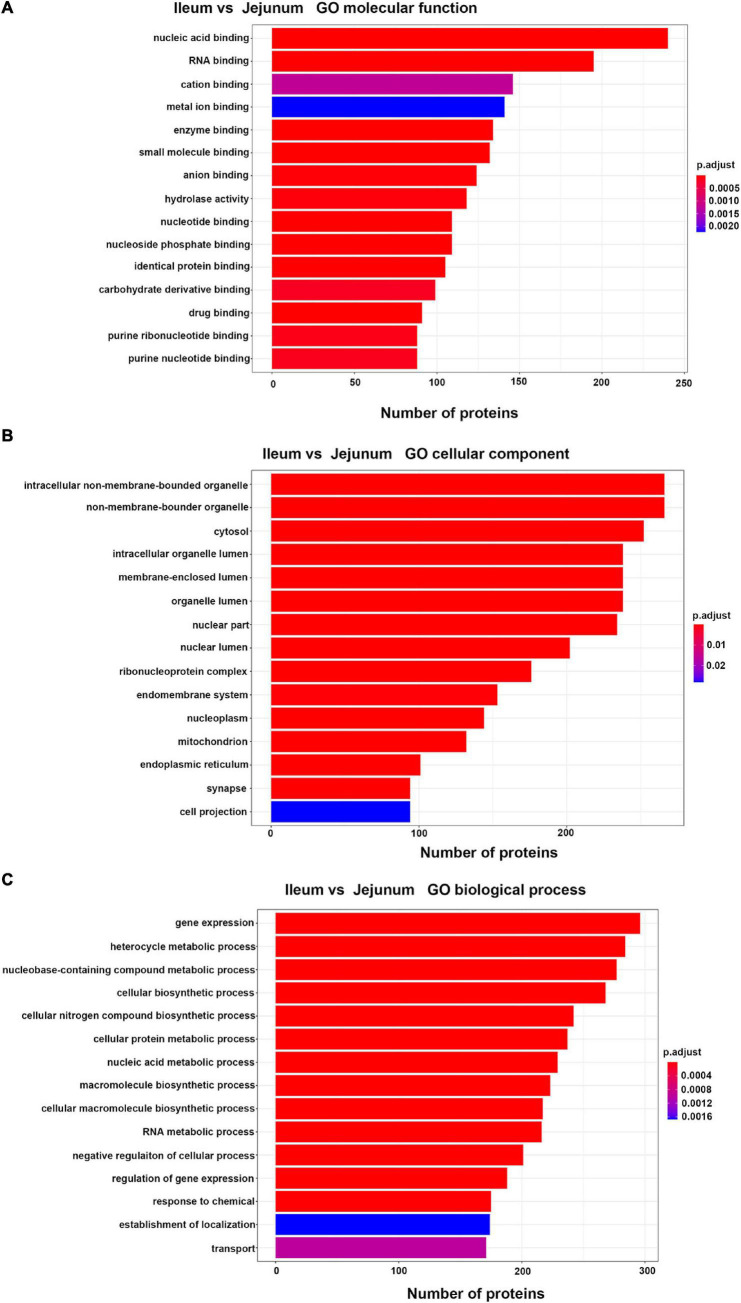
Top 15 of GO enrichment analysis in differentially expressed proteins between ileum and jejunum IEC-Exos. **(A)** GO molecular function analysis. **(B)** GO biological process analysis. **(C)** GO cellular component analysis.

Differentially expressed proteins between ileum and jejunum IEC-Exos were mainly involved in the molecular functions of binding and hydrolase activity, suggesting that these proteins were mainly related to biological functions through target cell fusion (Figure 3A). CC was mainly distributed in bound organelles, the cytosol, the organelle lumen, the nucleus, the ribonucleoprotein complex, and the endomembrane system, reflecting the formation of IEC-Exo proteins (Figure 3B). BP was mainly involved in gene expression, metabolic processes, biosynthetic processes, regulation processes and transport (Figure 3C).

#### Gene Ontology Analysis of Up-Regulated Proteins

**FIGURE 4:**
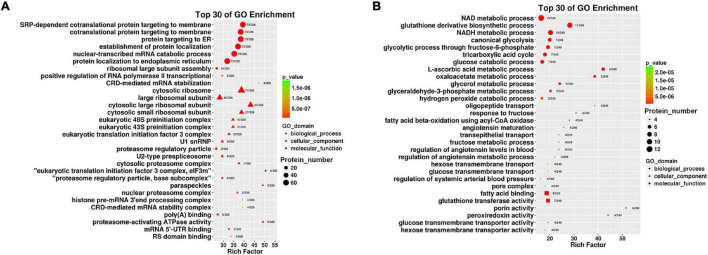
Top 30 of GO enrichment in up/down regulated proteins in IEC-Exos. **(A)** GO enrichment analysis of up-regulated protein. **(B)** GO enrichment analysis of down-regulated protein.

Up-regulated proteins were involved in poly (A) binding, proteasome-activating ATPase activity, mRNA5′-UTR binding, RS domain binding and other complex molecular functions. CC was mainly distributed in cytosolic ribosomes, the large ribosomal subunit and the small ribosomal subunit. BP was mainly involved in cotranslational protein targeting to the membrane, the endoplasmic reticulum, the establishment of protein localization, and nuclear-transcribed mRNA catabolic processes (Figure 4A). It was suggested that the differentially expressed proteins with high expression in IEC-Exos from the ileum were mainly involved in protein biosynthesis and processing modifications.

#### Gene Ontology Analysis of Down-Regulated Proteins

Down-regulated proteins were involved in fatty acid binding, glutathione transferase activity, porin activity, peroxiredoxin activity, the glucose transmembrane transporter, the hexose transmembrane transporter and other complex molecular functions. CC was mainly distributed in the pore complex. BP was mainly involved in NAD metabolic processes, NADH metabolic processes, glutathione derivative biosynthetic processes, glucose metabolism, the tricarboxylic acid cycle and glycerol metabolic processes (Figure 4B). It was suggested that the differentially expressed proteins with high expression in IEC-Exos from the jejunum were mainly involved in the metabolism of the three major nutrients, sugar, fat and protein, and the redox reaction (antioxidant effect, integrated detoxification effect).

### Kyoto Encyclopedia of Genes and Genomes Pathway Analysis

#### Kyoto Encyclopedia of Genes and Genomes Pathway Analysis of IEC-Exo Proteins From the Ileum and Jejunum

**FIGURE 5:**
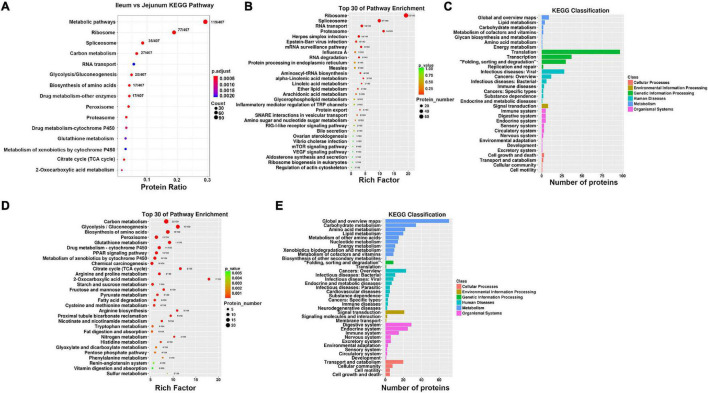
Enrichment analysis of KEGG pathway. **(A)** KEGG pathway analysis of differentially expressed proteins between ileum and jejunum IEC-Exos. **(B)** KEGG pathway enrichment analysis of up-regulated protein. **(C)** KEGG pathway classification of up-regulated protein. **(D)** KEGG pathway enrichment analysis of down-regulated protein. **(E)** KEGG pathway classification of down-regulated protein.

Differentially expressed proteins between ileum and jejunum IEC-Exos mediated metabolic pathways, ribosomes, spliceosomes, RNA transport and drug metabolism, etc. (Figure 5A).

#### Kyoto Encyclopedia of Genes and Genomes Pathway Analysis of Up-Regulated Proteins

The differentially expressed proteins with high expression in IEC-Exos from the ileum were mainly enriched in signaling pathways such as ribosome, spliceosome, RNA transport, and proteasome pathways (Figure 5B). The KEGG analysis results showed that these proteins were mainly involved in genetic information processing, including translation, transcription, folding, sorting, and degradation. These proteins mainly affected biological processes such as cell growth and death, lipid metabolism and signal transduction. In addition to being related to the function of the digestive system, these proteins were also related to the functions of the immune system, endocrine system, circulatory system and nervous system. In addition, these proteins were closely related to infectious diseases (viral or bacterial), cancers and immune diseases (Figure 5C).

#### Kyoto Encyclopedia of Genes and Genomes Pathway Analysis of Down-Regulated Proteins

The differentially expressed proteins with high expression in IEC-Exos from the jejunum were mainly enriched in signaling pathways such as metabolism, peroxisome, drug metabolism, and the renin angiotensin system (Figure 5D). The KEGG analysis results showed that these proteins were mainly involved in metabolic pathways, including carbohydrate metabolism, amino acid metabolism, lipid metabolism and other amino acid metabolism. These proteins mainly affected processes such as cell transport and catabolism, cellular immunity and signal transduction. In addition to being closely related to the function of the digestive system, these proteins were also related to the functions of the endocrine system, immune system, nervous system, excretory system, circulatory system, etc. Furthermore, these proteins were related to infectious diseases (bacterial, viral or parasitic), endocrine and metabolic diseases, cardiovascular diseases, cancers, immune diseases, neurodegenerative diseases and other diseases (Figure 5E).

### Protein–Protein Interaction Network

**FIGURE 6:**
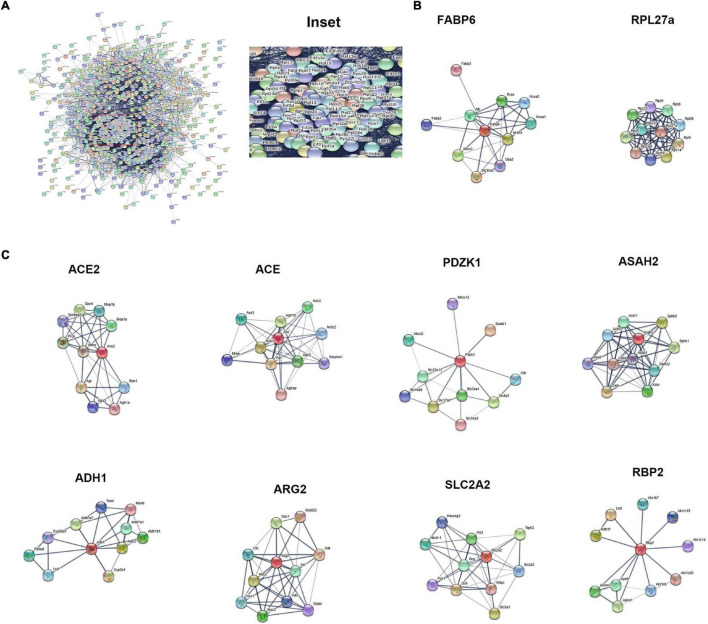
Protein–protein interaction network. **(A)** PPI network analysis of the hub of the differentially expressed proteins between ileum and jejunum IEC-Exos. **(B)** PPI network analysis of up-regulated proteins. **(C)** PPI network analysis of up-regulated proteins.

The hub of the differentially expressed proteins between ileum and jejunum IEC-Exos included ribosomal proteins (Figure 6A). The PPI network showed that the up-regulated proteins in ileum IEC-Exos were FABP6, Slc10a2, and RPL27a (Figure 6B), which are mainly involved in bile acid transport, fatty acid metabolism and protein synthesis pathways. However, the up-regulated proteins in jejunum IEC-Exos mainly included ACE2, ACE, Pdzk1, Asah2, ADH1, ARG2, Slc2a2, and Rbp2 (Figure 6C), which mediate sugar metabolism, fatty acid metabolism, amino acid metabolism, drug metabolism, bone metabolism, vitamin absorption, the renin-angiotensin system (RAS), NO, etc.

## Discussion

In this study, ileum and jejunum IEC-Exos were extracted by ultracentrifugation. Under transmission electron microscopy, both ileum and jejunum IEC-Exos presented as round or elliptical vesicles with a clear double-layered membrane structure, which conformed to the characteristics of exosomes. Subsequently, we detected the expression of the exosomal-specific marker proteins CD81 and TSG101 by western blotting. The results of nanoparticle tracking analysis showed that the diameter of ileum IEC-Exos was mainly concentrated at 30–130 nm, and the diameter of jejunum IEC-Exos was mainly concentrated at 30–120 nm, both of which were in the range of the diameter of exosomes.

Exosomes are rich in active substances, such as proteins, nucleic acids, and lipids. The proteins in exosomes play important roles in the processes of cellular material transport, signal transduction and antigen presentation. Exosomes derived from different cells have differences in the compositions and functions of their contents (Mathivanan et al., 2010; Pegtel and Gould, 2019). Proteomics studies on exosomes are of great significance for revealing the pathogenesis of related diseases, identifying biomarkers for disease diagnosis and prognosis, and screening disease treatment targets (Xiao et al., 2009; Kalluri and LeBleu, 2020).

In this study, for the first time, the iTRAQ quantitative proteomic approach combined with LC-MS/MS technology was used to detect IEC-Exo proteins, which was helpful for identifying exosomal proteins. The results showed that compared with jejunum IEC-Exos from ileum IEC-Exos, there were 393 up-regulated proteins and 346 down-regulated proteins, which indicated that differentially expressed proteins between ileum and jejunum IEC-Exos might perform different biological functions. Through analysis of biological information, we studied and compared the biological functions and related signaling pathways of the differential expressed proteins between ileum and jejunum IEC-Exos, which improved our understanding of the functions of the ileum and jejunum. Abundant exosomal proteins may have important significance in revealing the different biological functions between the ileum and jejunum.

The mammalian intestine is covered by a single layer of epithelial cells that is renewed every 4–5 days and performs the main functions of digestion and water and nutrient absorption. In addition, epithelial cells form a barrier against pathogens in the cavity. Due to the technological advancement of large-scale single-cell transcriptome profiling, more precise and comprehensive descriptions of cell types have been obtained from a multitude of organs.

Based on the expression of known marker genes in humans (Wang Y. et al., 2020) and mice (Haber et al., 2017), eight different cell types were identified, including enterocyte cells, goblet cells, Paneth cells, enteroendocrine cells, tuft cells, progenitor cells, transit amplifying (TA) cells and stem cells. In our study, the expression of IEC-Exo proteins was consistent with the abovementioned mRNA expression, indicating that various small intestinal epithelial cells can secrete exosomes. Furthermore, IEC-Exos contained a variety of cell-derived proteins and played an important role in regulating the biological functions of the intestine.

The small intestine is the main organ involved in nutrient digestion and absorption. Different sections of the small intestine have different digestion and absorption capacities for nutrient molecules (Borgstrom et al., 1957; Silk et al., 1974). Most of the digestion products of carbohydrates, proteins and lipids are absorbed in the duodenum and jejunum. When the nutrients in food reach the ileum, they are mostly digested and absorbed, so the ileum is the reserve part for absorption. However, the ileum has a unique role of actively absorbing bile salts and vitamin B12 and plays an important role in the digestion and absorption of fatty acids. Moreover, the small intestine is the main organ involved in oral drug absorption and drug metabolism (Billat et al., 2017). Studies have shown that small intestinal epithelial cells contain a large number of bioconverting enzymes. The longitudinal distributions of small intestinal cytochrome P450, glutathione S-transferase and bilirubin-uridine 5′-diphosphate (UDP)-glucuronyltransferase have a downward trend from the duodenum to the ileum (Tahir et al., 1988; Peters et al., 1989), suggesting that the antioxidant and detoxification effects of the proximal small intestine are stronger than that of the distal small intestine.

Intestinal epithelial cell derived exosomes selectively enrich protein bioactive components derived from small intestinal epithelial cells, which can regulate multiple functions and biological processes of cells after being taken up by target cells. Our study explored the MF, CC, and BP of differentially expressed proteins from ileum and jejunum IEC-Exos based on GO analysis. These proteins were mainly involved in gene expression, metabolic processes, biosynthetic processes, regulatory processes, and transport. The highly expressed differentially proteins – in ileum IEC-Exos were closely related to the transport of bile acids and the digestion and absorption of fatty acids. The level of participation of jejunum IEC-Exo proteins in of sugar, fat, protein, and drug metabolism and redox reactions was higher than that of ileum IEC-Exos, suggesting that the highly expressed differentially proteins in ileum and jejunum IEC-Exos played different biological roles, which was consistent with the aforementioned functions of the small intestine.

Kyoto encyclopedia of genes and genomes biological information analysis showed that the differentially expressed proteins between ileum and jejunum IEC-Exos were mainly involved in metabolic processes, the ribosome, the spliceosome, RNA transport and drug metabolism-other enzymes. The highly expressed differentially expressed proteins in ileum IEC-Exos were mainly enriched in genetic information processing pathways, which mainly affected processes such as cell growth and death, lipid metabolism and signal transduction. The highly expressed differentially expressed proteins in jejunum IEC-Exos were mainly enriched in metabolic pathways, which mainly affected cell transport and catabolism, cellular immunity, and signal transduction. These results indicated that the differentially expressed between the ileum and jejunum IEC-Exos participated in different signaling pathways and played important roles in regulating intestinal biological functions.

Ribosomal proteins and RNA form the ribosome, which is the organelle involved in protein synthesis. In addition to participating in protein synthesis, ribosomal proteins also have a wide range of *in vitro* functions in ribosomes, such as regulation of gene transcription, mRNA translation, cell proliferation, differentiation, and apoptosis (Warner and McIntosh, 2009; Zhou et al., 2015). In our study, PPI analysis showed that the core differentially expressed proteins between ileum and jejunum IEC-Exos included ribosomal proteins, which had the characteristics of active proliferation, strong self-renewing ability, and extensive biological functions of small intestinal epithelial cells. In particular, ileum IEC-Exos contained a variety of ribosomal proteins, suggesting that ileum epithelial cells participate in more physiological functions and pathophysiological processes than jejunum epithelial cells and have a stronger renewal speed and damage repair potential. Partial small bowel resection experiments performed in rats found (Ziegler et al., 1998) that the ileum exhibited a stronger adaptive growth ability than the jejunum, which was consistent with our results.

The core proteins FABP6 and SLC10A2, which were highly expressed in ileum IEC-Exos, are important bile acid transporters that are regulated by bile acids, cholesterol and hormones (Xiao and Pan, 2017). Dysfunctions of FABP6 and SLC10A2 are mainly involved in hepatobiliary diseases, inflammatory bowel disease, metabolic diseases and intestinal tumors. Thus, FABP6 and SLC10A2 are expected to become new targets for the treatment of related diseases and drug discovery (Zhang Y. et al., 2019; Yang et al., 2020). RPL27a is an important ribosomal protein that interacts with other ribosomal proteins to participate in protein synthesis. Clinical studies have found that RPL27a-related genes and pathways are closely related to the occurrence and development of intestinal tumors (Yajima et al., 2007; Yu et al., 2019). FABP6, SLC10A2, and RPL27a were highly expressed in ileum IEC-Exos, indicating that ileum IEC-Exos played key roles in bile acid transport, fatty acid digestion and absorption, and protein synthesis, which are associated with the occurrence and development of ileum functions and related diseases.

The SLC superfamily is one of the most important membrane transporter families in the cell membrane. The SLC superfamily is involved in essential physiological functions, such as intercellular substance transport, energy transfer, nutrition and metabolism, and signal transduction (Schumann et al., 2020). SLC transporters were found in jejunum IEC-Exos, indicating that they play an important role in the occurrence and development of material metabolism and related diseases, and are expected to become new therapeutic targets for the treatment of metabolic diseases.

Protein–protein interaction analysis further showed that ACE2, ACE, Pdzk1, Asah2, ADH1, ARG2, Slc2a2, and Rbp2 were enriched in jejunum IEC-Exos and participated in many signaling pathways. ACE2 and angiotensin-converting enzyme (ACE) are the key regulators of the RAS. The ACE2-Ang(1-7)-Mas receptor axis and ACE-Ang II-AT1 receptor axis are antagonistic to each other and interact with Mme, which plays an important role in regulating cardiovascular function, respiratory function, water and electrolyte balance, intestinal homeostasis, bone metabolism and nervous system function (Imai et al., 2010).

A novel coronavirus, SARS-CoV-2, has caused a global pandemic of COVID-19. Viral infection with SARS-CoV-2 causes a series of respiratory illnesses, including severe respiratory syndrome, indicating that the virus most likely infects respiratory epithelial cells and mainly spreads *via* the respiratory tract from human to human. However, gastrointestinal symptoms have been found in a substantial proportion of patients with COVID-19. Viral RNA has been detected in respiratory and stool specimens of patients, suggesting that SARS-CoV-2 may cause an enteric infection, in addition to a respiratory infection (Galanopoulos et al., 2020; Jin et al., 2021). Research has confirmed robust SARS-CoV-2 replication in human intestinal organoids, suggesting that the human intestinal tract may be a transmission route of SARS-CoV-2 (Zhou et al., 2020). It has been reported that ACE2 is the main host cell receptor of SARS-CoV-2 and that it plays a crucial role in the entry of virus into the cell to cause infection (Ziegler et al., 2020; Bickler et al., 2021). Furthermore, ACE2 expression has mainly been observed in human enterocytes, renal tubules, gallbladder, cardiomyocytes, vasculature and the lung (Hikmet et al., 2020). The distribution of viral receptors in different cell types of diverse tissues may indicate viral tropism and potential transmission routes, and SARS-CoV-2 productively infects human gut enterocytes (Lamers et al., 2020). IEC-Exos contained ACE2, which is new evidence that SARS-CoV-2 can infect intestinal cells.

Proteins, genomic molecules and receptors from infected cells make healthy cells more susceptible to infection. Exosome-mediated transfer of viruses may participate in viral infection (Pocsfalvi et al., 2020; Todd and Tripp, 2020) but has not yet been fully elucidated for coronaviruses. IEC-Exos may be a potential route of SARS-CoV-2 infection and may provide new ideas for further in-depth exploration of the mechanism of multiple organ damage in COVID-19. Jejunum IEC-Exos contained a higher level of ACE2 than ileum IEC-Exos, suggesting that the jejunum might be more susceptible to SARS-CoV-2. These findings provide a rich resource for future investigations of COVID-19 and its pathogenesis.

The small intestine is not only the main site of oral drug absorption but is also an important site of drug metabolism. The main site of intestinal absorption and metabolism of most oral drug formulations is the proximal small intestine because the proximal small intestine contains a large number of transporters and drug metabolism-related enzymes. The PDZ kinase 1 (PDZK1) protein is a member of a family of transporter adaptor proteins containing PDZ domains. The PDZK1 protein mainly binds to drug transporters and regulates their location, expression and function, which mediate the transmembrane transport of a variety of nutrients, endogenous substances and drugs (Sugiura et al., 2008; DeGorter et al., 2012). Jejunum IEC-Exos enriched a higher level of the PDZK1 protein than ileum IEC-Exos, providing further evidence that the jejunum plays a major role in drug absorption and metabolism.

The neutral ceramidase *N*-acylsphingosine amide hydrolase 2 (ASAH2) is a key enzyme in ceramide metabolism. ASAH2, which is expressed in the intestine, plays a major role in ceramide metabolism in the gut (Kono et al., 2006; Ohlsson et al., 2007), and ceramide mainly affects metabolic and disease states. An accumulation of ceramide has been observed in type 2 diabetes mellitus (T2DM), non-alcoholic fatty liver disease (NAFLD), inflammatory bowel disease (IBD), Alzheimer’s disease (AD), and cancer. Clarifying the role of ASAH2 in human diseases and determining its potential for use in the treatment of metabolic disorders and neurodegenerative diseases are critical (Parveen et al., 2019).

Traditionally, alcohol dehydrogenase 1 (ADH1) and aldehyde dehydrogenase 2 (ALDH2) in the liver are key enzymes in the ethanol metabolism pathway. Studies have shown that both ADH1 and ALDH2 are expressed in epithelial cells in the human digestive tract, especially in the proximal small intestine, which is the main site of ethanol metabolism (Chiang et al., 2012). ALDH1 (the ADH1 gene cluster: ADH1A, ADH1B, and ADH1C) plays a key role in the metabolic pathways of substances such as alcohol and retinol. Abnormal or unregulated expression of ALDH1 can cause liver disease, tumors, alcohol addiction and vitamin A absorption disorder (Gaviria-Calle et al., 2018; Wang S.C. et al., 2020; Sun et al., 2021). Jejunum IEC-Exos contained higher levels of ADH1, indicating that it plays an important role in ethanol metabolism and vitamin A absorption.

Arginase-2 (Arg-2) is expressed in the small intestine, kidney, brain, lactating mammary glands, monocytes, and macrophages (Kohler et al., 2008; Choi et al., 2012). Arg-2 and nitric oxide synthase (NOS) participate in the intracellular arginine metabolism pathway, regulating the intracellular concentration of arginine and the synthesis of NO, proline and polyamines in organisms. Furthermore, Arg-2 and NOS play important roles in regulating cardiovascular function, the inflammatory response, oxidative stress, immune function, and tumor occurrence and development. Inhibition of arginase is proposed as a method to improve antitumor immune responses (*via* activation and proliferation of T and NK cells) (Borek et al., 2020). Clinical studies have shown that L-Arginine/Nitric Oxide pathway was closely related to Crohn’s disease (CD) (Krzystek-Korpacka et al., 2020), CD can occur anywhere in the entire digestive tract, but is more common in the distal ileum and right colon. Compared with normal tissues, Arg-2 was down-regulated in the inflamed small intestine with CD. We found that IEC-Exos enriched Arg-2, especially ileum IEC-Exos contained lower levels of Arg-2, indicating that IEC-Exos may be involved in the pathogenesis of CD.

Slc2a2 (GLUT2) is expressed in small intestinal epithelial cells and mediates the glucose metabolism pathway, glucose absorption, and gluconeogenesis. In addition, GLUT2 participates in the glucagon and insulin signaling pathways, which are closely related to the occurrence of diabetes (O’Brien et al., 2018; Holman, 2020).

Retinol-binding protein 2 (Rbp2) is highly expressed in proximal small intestinal epithelial cells. The interactions between RBP2 and apolipoprotein are mainly involved in the binding, absorption, transport and metabolism of vitamin A and lipids (Blaner et al., 2020). Jejunum IEC-Exos contained high levels of RBP2, which could participate in the proliferation, migration, metastasis, and drug resistance of cancer cells. Thus, RBP2 may be a target for tumor treatment (Qi et al., 2014).

In addition to its important digestion and absorption functions, the intestine is also a very important immune organ and endocrine organ of the human body. Small intestinal epithelial cells synthesize and secrete a variety of cytokines to participate in the digestion and absorption of nutrients, microbial defense and immune responses, and endocrine functions. In our study, it was observed that the differentially expressed proteins enriched in ileum and jejunum IEC-Exos were not only closely related to the function of the digestive system but also related to infectious diseases, endocrine and metabolic diseases, cardiovascular diseases, cancers, immune diseases, neurodegenerative diseases and osteoarthritis. This study provides a new experimental basis for further in-depth study of the digestive system and disease occurrence.

## Conclusion

In summary, IEC-Exos contained a variety of cytokines secreted by intestinal epithelial cells, and there were many differentially expressed proteins between ileum and jejunum IEC-Exos, which played different roles in regulating intestinal biological functions. The highly expressed differentially expressed proteins in ileum IEC-Exos mainly mediated the functions of bile acid transport, fatty acid metabolism, protein synthesis and processing modifications. Moreover, the proteins in jejunum IEC-Exos mainly mediated sugar metabolism, fatty acid metabolism, amino acid metabolism, drug metabolism, bone metabolism, vitamin absorption, the RAS and the NO signaling system. IEC-Exos, especially in the jejunum, contained high levels of ACE2, which provided further evidence that SARS-CoV-2 infection could occur in the intestine. This study provided an important basis for further in-depth study of the function of small intestinal epithelial cells and related diseases. However, these results were approached in the manner of bioinformatics analysis; therefore, further verification is required.

## Data Availability Statement

The data presented in the study are deposited in the ProteomeXchange repository (http://www.proteomexchange.org/), accession number PXD030945.

## Ethics Statement

The animal study was reviewed and approved by 2020-0215.

## Author Contributions

ZD and CZ designed the research, performed the collection of exosome samples, and contributed to writing scripts. ZD, CZ, and BZ performed the proteome analysis and data analysis. QL supervised and guided the project. All the authors contributed to the article and approved the submitted version.

## Conflict of Interest

The authors declare that the research was conducted in the absence of any commercial or financial relationships that could be construed as a potential conflict of interest.

## Publisher’s Note

All claims expressed in this article are solely those of the authors and do not necessarily represent those of their affiliated organizations, or those of the publisher, the editors and the reviewers. Any product that may be evaluated in this article, or claim that may be made by its manufacturer, is not guaranteed or endorsed by the publisher.

## Footnotes

1
http://string.embl.de/
